# Treatment of chronic low back pain in patients with spinal deformities using a sagittal re-alignment brace

**DOI:** 10.1186/1748-7161-4-7

**Published:** 2009-03-09

**Authors:** Hans-Rudolf Weiss, Mario Werkmann

**Affiliations:** 1 Koob-Scolitech, Orthopedic Rehabilitation Services, Huehnerhof 100, D-55568 Abtweiler, Germany; 2 Orthomed Orthopedic Technical Services, Bad Sobernheim, Germany

## Abstract

**Background:**

For adult scoliosis patients with chronic low back pain bracing is initially indicated before spinal surgery is considered. Until recently there has been a lack of research into the effect upon pain reductions in the mid and long-term. Promising results have been documented in short-term studies for the application of a sagittal re-alignment brace in patients with spinal deformities and along with pain; however mid-term and long-term results are not yet available. The purpose of this study is to investigate the mid-term effects of this brace with respect to pain control.

**Materials and methods:**

67 patients (58 females and 9 males) with chronic low back pain (> 24 months) and the diagnosis of scoliosis or hyperkyphosis were treated with a sagittal re-alignment brace (physio-logic brace™) between January 2006 and July 2007. The indication for this kind of brace treatment was derived from a positive sagittal re-alignment test (SRT) and the exclusion of successful conservative treatment during the last 24 months. The aim of this type of conservative intervention was to avoid surgery for chronic low back pain.

**Results:**

The average pain intensity was measured on the Roland and Morris VRS (5 steps) before treatment. This was 3.3 (t1), at the time of brace adjustment it was 2.7 (t2) and after at an average observation time of 18 months it was 2.0 (t3). The differences were highly significant in the Wilcoxon test.

**Discussion:**

Short-term measurements showed that a significant pain reduction is possible in chronic postural low back pain using a sagittal re-alignment brace inducing lumbar re-lordosation. In a preliminary report at adjustment (t2), highly significant improvements of pain intensity have also been demonstrated. At 6 months of treatment however, no improvement was measured. The improvement of the mid-term effects (18 months) found in this study compared to the preliminary report may be due to the changed approach to compliance: whilst the bracing standard was not changed; the patients in this study were obligated to wear the brace for a minimum of 20 hrs per day for the first 6 months of treatment.

**Conclusion:**

The effect of the sagittal re-alignment brace leads to promising short-term improvements in patients with chronic low back pain and spinal deformities. Contrary to unspecific orthoses, which after a short period without persistent pain reduction are omitted by the patients, the sagittal re-alignment brace (physio-logic™ brace) leads to an effective reduction of pain intensity in mid-term even in patients who have stopped brace treatment after the initial 6 months of treatment. In conservative treatment of chronic low back pain specific approaches such as the sagittal re-alignment brace are indicated prior to considering the surgical options.

## Background

Loss of lumbar lordosis correlates well with the incidence of chronic low back pain in adulthood [1,2]. Sedentary lifestyle contributes to loss of lumbar lordosis as well as scoliosis and thoracolumbar or lumbar kyphosis. Therefore this condition should be addressed by physiotherapy and braces, which improve the sagittal mal-alignment and essentially re-establish lumbar lordosis.

For adult scoliosis patients with chronic low back pain bracing should be indicated before spinal surgery is considered, but until now no investigations have established the effects upon pain reductions in the mid or long-term application of these treatments.

The success rate of such a treatment in general does not appear to be high and compliance is generally described as moderate or poor [3-5], however a significant pain reduction has not been reported upon in the literature [5,6].

The treatment with the "sagittal re-alignment brace" [7] (physio-logic™ brace; EP 1 604 624 A1) has shown to improve chronic low back pain in a short test period [8]. As the brace impairs patterns of movement leading to a lumbar kyphosis, sitting activities require re-education, such as car driving with the brace. For these reasons the brace has been less favourable with the patient; however all of the patients appreciated the benefits of the immediate reduction in pain whilst in the brace. Thus a prospective study was carried out to estimate also mid-term effects of the treatment with the physio-logic™ brace.

The latest standard of this sagittal re-alignment brace was developed in summer 2006 from a prototype, first adjusted to an adult scoliosis patient with pain in 2004 [8]. Since then it has been applied in mature adolescents with scoliosis as the final brace applied before weaning; in a few patients at risk for progression where the application of a Chêneau brace has not been possible and for the treatment of chronic low back pain in scoliosis patients [8].

In adolescents with lumbar scoliosis, a curve correction has been demonstrated, showing that a 29° lumbar curve in a female patient with scoliosis, also suffering from Guillan Barré Syndrome, gained a correction to 19° in the brace. It would seem that structural thoracic curves can not be corrected in the mid-term just by using this small lumbar brace without additional lateral thoracic support.

In a preliminary report, involving scoliosis patients reporting pain, the physio-logic™ brace has proven to be effective at reducing their pain intensity, whilst the patients remained in the brace. The results seem superior, when compared to other brace types, usually applied for pain treatment as reported in international literature [8].

Additionally, in this study we found that this brace also effectively reduces back pain in non-scoliotic subjects.

The first mid-term results with patients followed up for a minimum of 6 months [9], were disappointingly unsuccessful.

In this first follow-up study [9], 29 Patients (females only) with an average Cobb angle of 37° (SD 22) and an average age of 41 years (SD 21) were treated with the physio-logic™ brace for an average of 7.5 Months (SD 5,6). The amount of allocated in-brace time as well as total in-brace time daily remained the individual patients' choice, however all patients agreed to wear the brace for a minimum of 8 hrs per day. Pain intensity was high (3,38) before treatment (scale 0–5) and low (1,28) after adjustment. The differences where highly significant in the Wilcoxon test (z = -4,29; p < 0,0001). After a follow-up of a minimum 6 months, the pain intensity was moderate to severe (2,69). The difference to the pre-treatment value was of no greater significance.

Although the brace seems to work in the short-term, the mid-term results according to the cited preliminary report [9] did not justify a prescription.

Compliance in the preliminary follow-up study [9] was poor, as in other studies about brace treatment in patients with low back pain [3-5]. The reasons for this may or may not relate to the individual patients' choice over brace wearing times.

Therefore [from 2006 onwards] the guideline given to the patients was altered accordingly:

The brace should be worn for at least 20 hours per day within the first 6 months. After that time (1) it can be expected, that the sagittal deformity has been mobilised enough to gain the desired functional improvement and (2) for the patient to have learnt to cope with the brace during activities of daily living and the impairment of posture and movement whilst wearing the brace. The expectation provided to the patient was that after the initial 6 months the patient may decide by himself to have a first brace-free interval, if the result finally was a consistent pain relief without the brace.

If pain increased in the brace-free interval, the initial procedure of brace treatment would re-start once again.

The purpose of this study was to investigate as to whether a change in the approach to the patients' education would improve the compliance in order to allow better treatment results in the mid-term. In the latter case a prescription of this brace would be justified in order to attempt to prevent surgery.

## Methods

**Figure 1:**
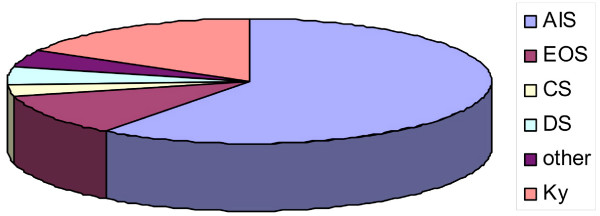
**Distribution of the diagnoses in the sample of patients**. There were patients with scoliosis and kyphosis in the sample described. 41 had an Adolescent Idiopathic Scoliosis (AIS), 4 had a Juvenile Idiopathic Scoliosis (Early onset scoliosis = EOS), 3 had an Infantile Idiopathic Scoliosis (EOS), 2 had congenital scoliosis (CS), 3 had scoliosis in combination with other diseases, 3 had degenerative (de novo) scoliosis (DS) and 11 patients had kyphosis (Ky).

67 patients (58 females and 9 males) with chronic low back pain (> 24 months) and the diagnosis scoliosis (n = 56; average scoliosis angle 41° ranging from 10 to 91°) or kyphosis (n = 11) were treated with a sagittal re-alignment brace (physio-logic brace™) between January 2006 and July 2007. The distribution of diagnoses can be seen in figure 1. The indication for this kind of brace treatment was derived from a positive sagittal re-alignment test (SRT, see [8]) and from the exclusion of any successful conservative treatment within the last 24 months. The main aim of this intervention was to measure reductions in pain and to avoid surgery for chronic low back pain.

Before brace prescription the brace action was simulated and pain reduction was tested [8]. Brace treatment was prescribed only when a significant reduction of pain intensity registered whilst performing the SRT [8] prior to bracing (Pain reduction of at least 2 steps on the VRS). Pain intensity was recorded before brace treatment (t1), within the 1^st ^week after brace adjustment (t2) and at follow-up, at average 18 months after brace adjustment (t3) using the Roland and Morris VRS [10].

### The brace

**Figure 2:**
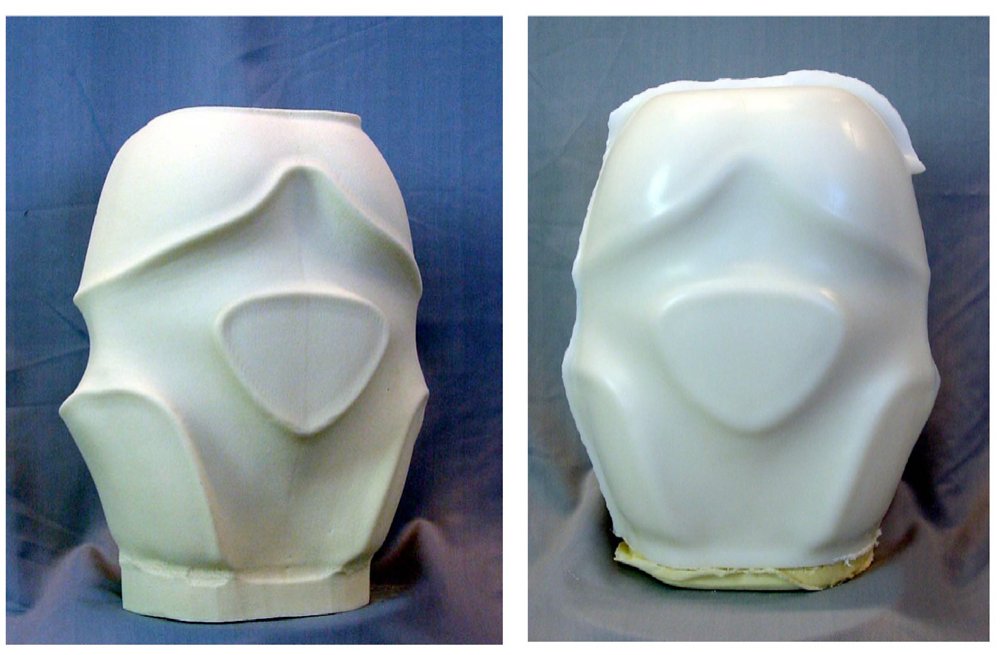
**Foam model of the physio-logic™ brace**. After the anthropometrical data (circumferential and longitudinal trunk measurements) are recorded, the foam model is milled from a blank hard foam block. This hard foam model is wrapped in a heated PE-plate, which is vacuumed to the models surface. The brace parts are cut from the PE-model and adjusted to the patient. The physio-logic™ brace is manufactured by Orthomed OT Services, Bad Sobernheim, Germany.

The physio-logic™ brace (Figure 2.) is manufactured using CAD technique as provided by Orthomed OT Services, Bad Sobernheim, Germany.

**Figure 3:**
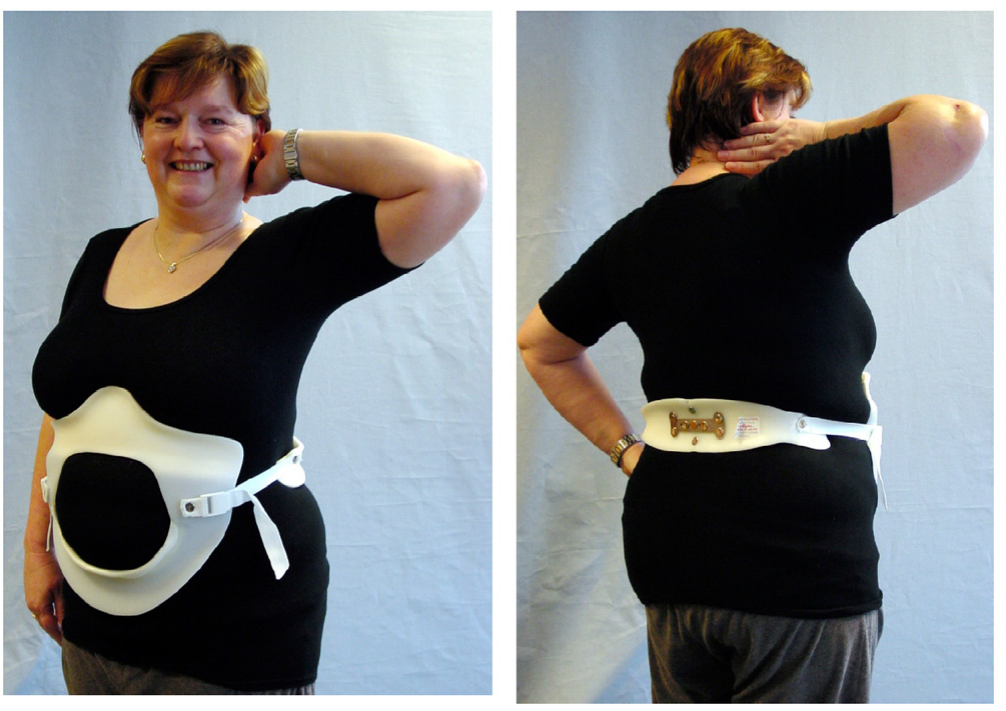
**Patient wearing a physio-logic™ brace**. When the brace is adjusted to the patient the main dorsal pressure should restore lordosis at the level of L2. Free space is necessary at the lower rib-bows while the pressure areas ventrally are located directly caudal of the pectoral region and cranial to the pubis [14]. (The patient provided her consent to be featured in the manuscript.)

After the anthropometrical data (circumferential and longitudinal trunk measurements) are registered, the foam model is milled from a blank hard foam block. This hard foam model is wrapped in a heated PE-plate, which is vacuumed to the models surface. The brace parts are cut from the PE-model and adjusted to the patient [7-9] (Figure 3.).

In principle the brace provides a crosswise ventral pressure area of 3 cm directly sub-pectoral, caudally followed by a void. The caudal ventral pressure point is supra-pubical. The dorsal pressure zone is situated at the waist level allowing an apical pressure at the level of L2. This dorsal pressure leads to a mass movement also ventralising the lower ribs into the ventral void of the brace. As there is a slight compression of the trunk unavoidable, lateral voids are necessary to give room for the shifted regions of the trunk there as well. If these voids are omitted the compression forces generated will inhibit breathing and make brace wearing impossible.

### The Questionnaire

The Roland and Morris questionnaire used for this study is a verbal rating scale (VRS) providing certain steps of subjective pain judgements recorded by the patients themselves [10]. It contains five rating steps from "low pain (1)" to "the pain is hardly to bear (5)"

### Modalities of brace wearing

While the bracing standard was not largely changed in comparison to the preliminary series [9], the patients in this study were obligated to wear the brace for a minimum of 20 hours per day at least for the first 6 months of treatment. After this period, brace wearing times were set by the individual patient, depending on the residual pain intensity.

### Definition of follow-up periods

Short-term = directly upon application to 14 days

Mid-term = 6 months to 24 months of follow-up

Long-term = > 24 months of follow-up

## Results

The average pain intensity recorded on the Roland and Morris VRS (5 steps) before treatment was 3.3 (t1), at the time of brace adjustment was 2.7 (t2) and after an average observation time of 18 months was 2.0 (t3). The differences were highly significant in the Wilcoxon test (see table 1).

**Table 1:** Statistical data for the whole group of patients

	Z	P1	P2
t1/t2	- 4,21416	< 0,001	< 0,001

t1/t3	- 5,85398	< 0,001	< 0,001

Comparison of the pain intensity as estimated with the help of the Wilcoxon test at t1 (before treatment)/t2 (at brace adjustment) and at t1 (before treatment)/t3 (after an average follow-up time of 18 months).

A group of 21 patients were able to completely remove the brace after significant improvements of pain intensity were recorded (see table 2). In certain patients the symptoms of spinal claudication were also significantly reduced.

**Table 2:** Statistical data for the group of 21 patients, who stopped bracing at 6 months of treatment.

	Z	P1	P2
t1/t2	- 1,76505	0,03877	0,07755

t1/t3	- 5,85398	0,00166	0,00333

Comparison of the pain intensity as estimated with the help of the Wilcoxon test at t1 (before treatment)/t2 (at brace adjustment) and at t1 (before treatment)/t3 (after an average follow-up time of 18 months). No significant differences t1/t2, but significant differences in the mid-term (t1/t3).

## Discussion

The role of the sagittal profile in the development of a lumbar scoliosis is not yet clear [11]. Correction of the lumbar sagittal profile in a sagittal re-alignment brace leads to an improvement of 3D correction and can in short-term, be compared to the correction effect of other larger braces aiming at a 3D correction [12]. It is necessary to recognise that the severity of symptoms in patients with back pain, as they increase in a linear fashion with progressive sagittal imbalance [1,2]. The results of these studies also show that hyperkyphosis is more favourable in the upper thoracic region but very poorly tolerated in the lumbar spine [1,2].

This is why the physio-logic™ brace may be regarded as the 'gold standard' for bracing of patients with pain in combination with a reduced lumbar lordosis, as the physiological sagittal profile is improved or even restored when this specific brace is applied.

In the mid-term a significant pain reduction is possible in chronic postural low back pain [13] using a sagittal re-alignment brace inducing lumbar re-lordosation, in a population whom otherwise may have chosen surgery.

In a preliminary report at adjustment (t2) highly significant improvements of pain intensity have been shown, however at 6 months of treatment no significant improvement was retained [9].

The improvement of the mid-term effects in this study, when compared to the preliminary report, may be due to the change in the approach patient education. Whilst the bracing standard was not largely changed, the patients were obligated to wear the brace at minimum 20 hours per day for the first 6 months. After this period the individual patients decided upon the brace wearing times by themselves. With this new approach to compliance we can predict a successful outcome using the sagittal re-alignment brace (physio-logic™ brace) in this specific patient population.

The brace should not be used in patients with chronic low back pain due to lumbo-sacral instability (e.g. spondylolisthesis), even if the SRT measured a pain reduction [13]. As with some cases a secondary increase in pain intensity is possible. To exclude the condition of a spondylolisthesis before brace application, X-rays of the lumbar spine to view the lumbo-sacral junction from the side are necessary.

**Figure 4:**
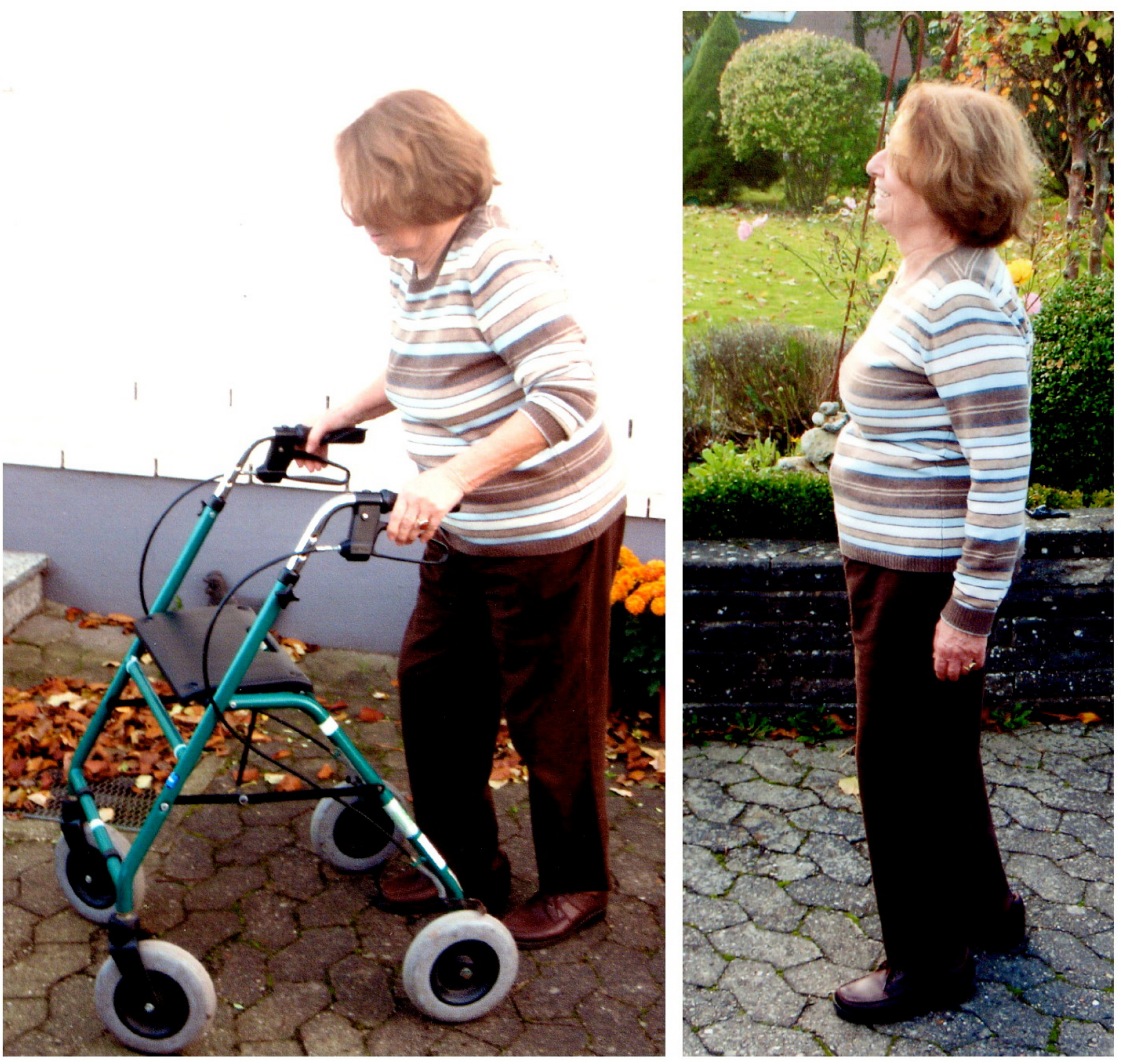
**Patient with chronic low back pain and neuromuscular scoliosis**. The patient was unable to walk in an upright posture without the brace. She needed a special aid for ambulation *(see picture on the left)*. Whilst in the brace she could omit this walking aid and has significantly less back pain *(as can be seen on the right)*. A constant relief of muscle tension has also been reported by this patient, who feels far less fatigue at the end of the day. (The patient provided her consent to be featured in the manuscript.)

The beneficial effects of this brace in patients with lumbar scoliosis and the symptoms of spinal claudication has also been demonstrated [14]; therefore lateral listhesis and spinal claudication should not be regarded as being contraindicated for this treatment. In contrary, when there is evidence that sagittal re-alignment leads to a correction of scoliosis [8,12,15], we may assume that this brace restores stability of the lumbar spine by locking the lumbar facet joints. As has been shown in several patients within this study, the restoration of function activity such as upright walking without the need of additional aids has been accomplished (Figure 4.).

## Conclusion

The effects of the sagittal re-alignment brace leads to promising short-term improvements in patients with chronic low back pain and spinal deformities. Contrary to unspecific orthoses, which after a short period without persistent pain reduction are omitted by the patients, the sagittal re-alignment brace (physio-logic™ brace) leads to an effective reduction of pain intensity at 18 months even in patients who have stopped brace treatment after the initial 6 months of treatment. Therefore in conservative treatment of chronic low back pain, specific approaches such as the sagittal re-alignment brace are indicated initially that aim to prevent spinal surgery.

## Competing interests

HRW is a consultant for Koob-Scolitech. MW does not declare any competing interests related to this paper.

## Authors' contributions

HRW contributed to patient acquisition, manuscript writing and statistics. MW contributed to patient acquisition and technical support.
